# New Insights into the Role of Autophagy in Tumor Immune Microenvironment

**DOI:** 10.3390/ijms18071566

**Published:** 2017-07-19

**Authors:** Chia-Jung Li, Wan-Ting Liao, Meng-Yu Wu, Pei-Yi Chu

**Affiliations:** 1 Research Assistant Center, Show Chwan Memorial Hospital, Changhua 500, Taiwan; nigel6761@gmail.com; 2 Chinese Medicine Department, Show Chwan Memorial Hospital, Changhua 500, Taiwan; enolainsky@gmail.com; 3 Graduate Institute of Chinese Medicine, China Medical University, Taichung 404, Taiwan; 4 Department of Emergency Medicine, Taipei Tzu Chi Hospital, Buddhist Tzu Chi Medical Foundation, New Taipei City 231, Taiwan; skyshangrila@gmail.com; 5 Department of Pathology, Show Chwan Memorial Hospital, Changhua 500, Taiwan; 6 School of Medicine, College of Medicine, Fu-Jen Catholic University, New Taipei City 242, Taiwan; 7 National Institute of Cancer Research, National Health Research Institutes, Tainan 704, Taiwan

**Keywords:** autophagy, tumor immunity, tumor microenvironment, tumor immunotherapy

## Abstract

The tumor microenvironment is a complex system that is affected by various factors, including hypoxia, acidosis, and immune and inflammatory responses, which have significant effects on tumor adhesion, invasion, metastasis, angiogenesis, and autophagy. In this hostile tumor microenvironment, autophagy of tumor cells can promote tumor growth and metastasis. As autophagy is a double-edged sword in tumors, treatment of cancer via regulation of autophagy is extremely complicated. Therefore, understanding the relationship between tumor autophagy and the tumor microenvironment is extremely important. As the immune milieu plays an important role in tumor development, immunotherapy has become a promising form of cancer therapy. A multi-pronged treatment approach using immunotherapy and molecular targets may become the major direction for future cancer treatments. This article reviews existing knowledge regarding the immune factors in the tumor microenvironment and the status of tumor autophagy research.

## 1. Molecular Regulatory Mechanisms of Autophagy

Autophagy is a biological process whereby large molecules and damaged organelles in the cytoplasm are degraded. A key step during this process is the formation of autophagosomes. Autophagosomes mainly consist of cytoplasmic contents and damaged organelles, such as the mitochondria. Depending on where the membranes of autophagosomes are generated from, the routes by which the contents in autophagosomes are transferred to lysosomes can be different [1]. Autophagy can be largely divided into three types. The first type is macroautophagy: membranes are derived from the endoplasmic reticulum and Golgi bodies by invagination to form a cup-shaped, double-membraned structure. This structure completely envelops the autophagy target to form an autolysosome, and the target is degraded by lysosomal enzymes [2,3]. The second form is microautophagy: here, the lysosome directly envelopes its target, and digestion occurs inside the lysosome. The last form of autophagy is chaperone-mediated autophagy (CMA): a chaperone protein first binds to the target protein to guide it to the lysosome, and the target proteins are then degraded by enzymes. The substrates involved in the CMA route are soluble protein molecules. However, not all soluble proteins can be degraded. Therefore, the CMA route demonstrates selectivity, which is a major difference from the other two forms of autophagy [4,5].

Misfolded proteins inside the body are mainly degraded by proteasomes and lysosomes; small proteins can be degraded by both proteasomes and lysosomes [6]. However, protein polymers that are larger in size require polyubiquitination markers before they can be degraded by autophagosomes [7]. However, regardless of whether degradation products pass through the proteasomal or the lysosomal route, they will ultimately all undergo autophagy-mediated degradation. Using macroautophagy as an example, autophagy can be largely divided into four main phases. The first step is formation of the isolation membrane. Under stress or nutrient-deprived conditions, the endoplasmic reticulum invaginates to form a cup-shaped, double-layered sequestering membrane, which begins to surround the target to form a phagopore. This is followed by the formation of autophagosomes. During this process, the isolation membrane continues to extend, and completely envelops the target and the surrounding cytoplasm to form an autophagosome. The third phase consists of transportation and fusion of autophagosomes. After formation of the autophagosome, it fuses with a lysosome, and the sequestered contents are transferred to the newly-formed autophagolysosome. Lastly, the target is degraded via acidification; when the pH has reached the required value inside the vesicle, the target is degraded by multiple lysosomal enzymes. The small molecules produced in this process can be recycled for further use in the cell [1,6].

**Figure 1:**
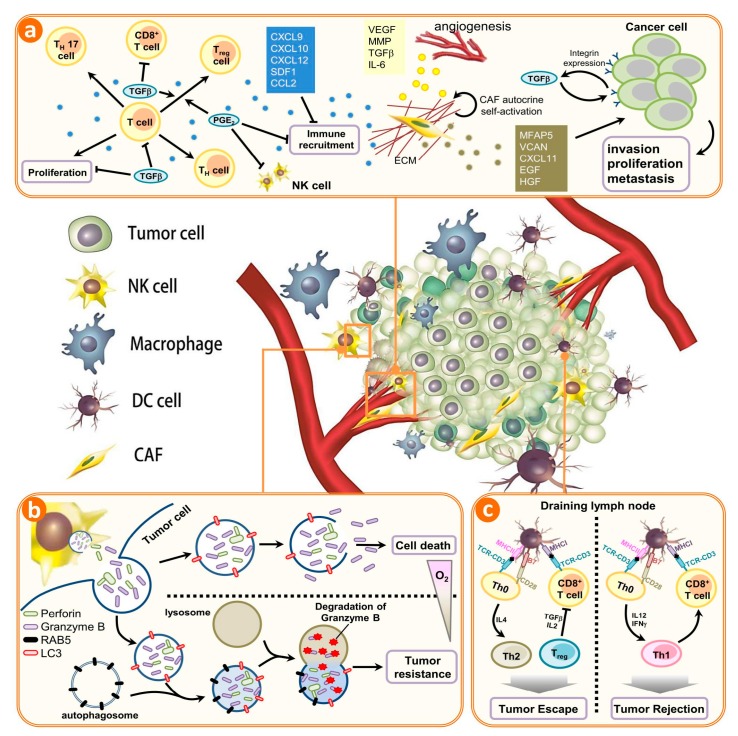
The crosstalk between autophagy and tumor immune microenvironment. A plethora of noncancerous cells in the tumor microenvironment regulate the infiltration, accumulation, and proliferation of immune cells in tumors. The immune system can be implicated in both inherent and acquired resistance to targeted therapies. (**a**) Cells of the innate and adaptive immune systems typically act to eliminate transformed and malignant cells. Rare tumor cells evade immune surveillance mechanisms and establish a microenvironment that stimulates tumor growth, proliferation, and angiogenesis. This is primarily mediated by tumor- and stromal cell–derived growth factor and cytokines that suppress the immune system while promoting tumor cell proliferation, angiogenesis, and metastasis. Under these conditions, factors secreted by immune effector cells recruited to the tumor site may contribute to tumor development. Tumor formation involves the co-evolution of neoplastic cells together with extracellular matrix and vascular endothelial, stromal and immune cells. The immune infiltrate can include multiple cell types, these cell populations can have both pro- and anti-tumor functions and can vary in their activation status and their localization within the tumor. The extracellular matrix (ECM), together with cellular components of the tumor microenvironment, are actively remodelled and reprogrammed by CAFs. CAFs can have significant plasticity and diverge with regard to activation status, localization within the tissue, stress response and origin. CAFs have multiple functions in the TME, in part through ECM-mediated T cell trapping and cytokine-regulatory T cell exclusion; (**b**) In normoxic cells, perforin forms pores in the gigantosome membrane, allowing granzyme B release and initiation of autophagy. In hypoxic cells, excessive autophagy leads to fusion of gigantosomes with autophagosomes and the subsequent formation of amphisomes, which contain granzyme B and perforin. Fusion of amphisomes with lysosomes triggers selective degradation of granzyme B, making hypoxic tumor cells less sensitive to natural killer (NK) cell–mediated killing; (**c**) Tumor cells show a decrease in the cell-surface levels of major histocompatibility complex (MHC) that is often associated with less antigen presentation; thus, there is reduced recognition and eradication of tumor cells by CD8^+^ T cells specific for conventional tumor antigens. However, immune targets can be divided into those that prime DC, those that affect T cell checkpoint co-stimulation, those that affect T cell exhaustion and those that affect T cell recruitment. CAFs, cancer-associated fibroblasts; NK, natural killer; DC, dendritic cell; PGE2, prostaglandin E2; TGFβ, transforming growth factor-β. CXCL, chemokine (C-X-C motif) ligand 1; SDF1, stromal cell-derived factor 1; CCL2, chemokine (C-C motif) ligand 2; VEGF, vascular endothelial growth factor; MMP, matrix metalloproteinases; IL-6, Interleukin 6; MFAP5, microfibrillar associated protein 5; VCAN, Versican; EGF, epidermal growth factor; HGF, hepatocyte growth factor; ECM, extracellular matrix; RAB5, Ras-related protein; LC3, light chain 3, MHC, major histocompatibility complex.

Autophagy is induced in cells during conditions such as starvation, hypoxia, chemotherapy, and oxidative stress [7]. The degraded and recycled cytoplasmic components can provide amino acids and adenosine triphosphate (ATP) to maintain protein synthesis and other necessary metabolic functions. Autophagy is regulated by autophagy-related proteins, which are encoded by autophagy-related genes (*Atg*) [8]. In mammals, autophagy is mainly induced by co-regulation of various stimuli and is initiated when *Atg13* and *RB1* inducible coiled-coil 1 (*RB1CC1*) form a unc-51 like autophagy activating kinase 1 (ULK1)–Atg13–RB1CC1–Atg10 complex that interacts with mammalian target-of-rapamycin complex 1 (mTORC1) [9,10]. Under nutrient and energy-deprived conditions, mammalian target-of-rapamycin (mTOR) activity is inhibited. mTOR is a serine/threonine protein kinase that belongs to the phosphatidylinositol 3 kinase-related kinase (PIKK) family. The activity of mTOR is inhibited under nutrient starvation, which is known to be a crucial step for autophagy induction in eukaryotes [11]. On the other hand, activation of ULK1, which regulates Atg13 and RB1CC, induces the formation of the autophagosome membrane. Extension of the autophagic vesicle requires the participation of two ubiquitin-like conjugation systems, Atg12–Atg5–Atg16 complex and Atg8/light Chain 3 (LC3) [12]. The yeast *Atg8* gene is homologous to the mammalian gene and is also termed LC3-I. Initially, free LC3-I in the cytoplasm binds to PE and undergoes lipidation to form LC3-II, which is localized on the outer membrane of the autophagosome. LC3-II is a specific target for autophagosome formation and is often used as a marker for autophagy induction. Autophagosomes are degraded by lysosomal enzymes. The signaling pathways that regulate autophagy include mTOR, phosphatidylinositol-4,5-bisphosphate 3-kinase (PI3K)-protein kinase B (Akt), p53, AMP-activated protein kinase (AMPK), and endoplasmic reticulum (ER) stress [13,14]. mTOR is downstream of the PI3K-Akt signaling cascade and regulates cell growth and proliferation. As it inhibits the initial stages of autophagy, inhibition of mTOR can induce autophagy [15] (Figure 1b). However, almost all of the factors involved in recruiting the ribosome, including *eIF4E* binding protein (4EBP), are phosphoproteins whose phosphorylation states are the best-characterized substrates of mTORC1, which promote protein synthesis and directly proportional to the growth rates of the cell [11].

There is a molecular mechanism for autophagy. Specialized molecules, such as kinases and enzymes that can bind and hydrolyze guanosine triphosphate (GTPases), participate in this process, all encoded by autophagy-related (*Atg*) genes (Table 1).

## 2. Functions of Autophagy in Tumors

In normal cells, autophagy inhibits the accumulation of reactive oxygen species (ROS) and removes damaged organelles in order to maintain genome stability and inhibit oncogenesis [16]. Beclin is an important regulatory factor for formation of autophagosomes [17]. Studies have shown that Beclin-1 is a tumor suppressor, and that the anti-apoptotic B-cell lymphoma 2 (Bcl-2) protein directly binds to the Bcl-2 homology 3 (BH3) domain of Beclin-1 to inhibit autophagy [18]. Mice deficient in Beclin-1 were found to be more prone to precancerous lesions of lymphomas, breast cancer, liver cancer, and lung cancer. These research findings all provide indirect evidence for the tumor suppression role of autophagy [19,20,21,22,23]. Inhibiting mTORC1 signaling has therefore attracted great attention as an anti-cancer therapy. However, progress in using inhibitors of mTOR signaling as therapeutic agents in oncology has been limited by a number of factors, including the fact that the classic mTOR inhibitor (rapamycin) inhibits only some of the effects of mTOR, the existence of several feedback loops, and the crucial importance of mTOR in normal physiology [24]. In addition, breast cancer 1 (*BRCA1*) is a tumor suppressor located close to *Beclin-1* on the genome. It is known that *BRCA1* mutations can induce the development of breast and ovarian cancers [18,25]. However, recent studies suggest that loss of *Beclin-1* may be a passenger mutation, since loss of *BRCA1* and *Beclin-1* itself does not have an effect on tumor suppressor functions [26].

It has been shown that tumor cells are usually in a hypoxic state and are associated with nutrient and growth factor deficiencies, which are activators of autophagy. Autophagy promotes the survival of tumor cells by providing the required nutrients. Therefore, induction of autophagy may be a form of self-preservation mechanism for tumor cells to survive in the hypoxic, highly acidic, and/or toxic (due to chemotherapy) environment [27,28,29]. Currently, cancer therapies usually consist of a combination of surgery and radiochemotherapy [30]. However, tumor cell resistance to chemotherapeutic agents results in tumor relapse following treatments. Chemotherapuetic agents mainly kill cancer cells by induction of apoptosis. However, the tumor microenvironment can regulate autophagy and senescence pathways to inhibit apoptosis, thus promoting survival of tumor cells and resistance to chemotherapy [31,32]. Therefore, autophagy plays diverse roles during different stages of tumor development [12,33,34]. Currently, the study of autophagy regulation and its molecular mechanisms in tumors has become an important focus in cancer research.

## 3. Tumor Immune Microenvironment

The tumor microenvironment (TME) includes resident stromal components, such as cancer cells, fibroblasts and endothelial cells, and recruited immune cells. In addition to the complex tissue architecture of a tumor, cancer cells within a single tumor are heterogeneous in their molecular signatures; this is referred to as intra-tumor heterogeneity. The TME-released chemotactic factors are responsible for leukocyte recruitment and activation. A better understanding of the role of these signals will provide insights into the development of new therapeutic approaches.

### 3.1. IL-1

The interleukin 1 (IL-1) family is a group of 11 cytokines, and the two main pro-inflammatory cytokines are IL-1α and IL-1β, which bind to the IL-1 receptors (IL-RI and IL-RII); the receptor antagonist for IL-1 is IL-1RA. IL-1 can inhibit signaling pathways such as cyclooxygenase (COX-1), phosphorylated inhibitor of κB (IκB), and stress-activated protein kinase/c-Jun N-terminal kinases (SAPK/JNK), thereby promoting tumor development, growth, and metastasis. Inhibition of IL-1 expression in tumor cells can induce upregulation of p21 and p53, leading to suppression of tumor growth [35]. Recent studies have shown that when the IL-1 pathway is inhibited in melanomas, LC3-II expression and the LC3-II/LC3-I ratio increased in tumor cells. In addition, the proportion of cells containing acidic vesicular organelles was also increased. This suggested that inhibition of the IL-1 pathway can induce autophagy in tumor cells and inhibit tumor growth [36,37].

### 3.2. Interferon-γ (IFN-γ)

This cytokine is produced by activated CD4 and CD8 T-cells, as well as natural killer (NK) cells, and can induce autophagy in various cell types including endothelial cells and hepatocytes. Similarly, in tumors, IFN-γ can induce epithelial cells, immune cells, and tumor cells to undergo autophagy. Studies have shown that IL-1β induces gastric cancer in mice through translocation of the H^+^/K^+^ ATPase-IFN-γ gene, and that IFN-γ is associated with tumor occurrence and autophagy [38]. Results indicated that overexpression of IFN-γ can inhibit the development of gastric mucosal tumors in mice. Furthermore, it can also inhibit autophagy in bone epithelial cells, which may be due to Beclin-1 regulation [39,40].

### 3.3. Macrophage Migration Inhibitory Factor (MIF)

This is a multi-functional cytokine with tautomerase activity. MIF plays important roles in inflammation regulation, cell proliferation, angiogenesis, tumor formation, and can also participate in tumor regulation. When recombinant MIF (rMIF) was expressed in human liver cancer cells, an increase in cytoplasmic LC3-I to LC3-II conversion was observed. Conversely, treatment with MIF-specific inhibitor resulted in inhibition of LC3-II conversion. When PI3K and ROS inhibitors were used to treat cells that overexpressed rMIF, conversion of LC3-I was similarly inhibited. Interestingly, both endogenous and exogenous MIF can induce ROS synthesis and decrease mitochondria membrane potential; inhibition of MIF shows the opposite effect. In addition, inhibition of MIF expression via shRNA resulted in a reduction in autophagy in liver cancer cells. Results from previous studies showed that under oxidative stress and during inflammation, MIF-induced autophagy in cells is associated with increased ROS production and activation of PI3K signaling. At the same time, a number of inflammatory factors in the tumor microenvironment such as IL-2, IL-6, IL-8, IL10, macrophage inflammatory protein 1 α (MIp1α), IFN-β, Transforming growth factor β (TGF-β), regulated on activation, normal T cell expressed and secreted (RANTES), and granulocyte-macrophage colony-stimulating factor (GMCSF) participate in regulation of autophagy in tumor cells [41,42].

### 3.4. Receptor for Advanced Glycation End Products (RAGE)

The RAGE is a member of the cell surface immunoglobulin family, and its ligands are High mobility group box 1 (HMGB1), as well as some proteins in the S100 protein family [43]. The interactions between RAGE and its ligands regulate cell survival and inflammatory responses mainly by inducing the expression of extracellular signal–regulated kinases (ERK) and nuclear factor κ-light-chain-enhancer of activated B cells (NF-κB) p65. In tumors, RAGE can enhance inflammation-associated carcinogenesis, leading to tumor formation. It can also increase the resistance of tumor cells to chemotherapy. When RAGE is deficient, apoptosis in tumor cell is increased while inflammatory responses are decreased. Recent studies have found that under oxidative stress, RAGE expression is increased, while mTOR phosphorylation is inhibited; the formation of Beclin1/vacuolar protein sorting 34 (VPS34) autophagosomes results in protective effects on tumor cells [44,45]. Targeted knockdown of RAGE in the tumor cell, leads to increased apoptosis, diminished autophagy and decreased tumor cell survival. In contrast, overexpression of RAGE is associated with enhanced autophagy, diminished apoptosis and greater tumor cell viability. RAGE limits apoptosis through a p53 dependent mitochondrial pathway. Moreover, RAGE-sustained autophagy is associated with decreased phosphorylation of mTOR and increased Beclin-1/VPS34 autophagosome formation [45].

### 3.5. Neutrophils

The IgA Fc receptor FcαRI (CD89) is a Fc receptor for IgA, and is involved in transmitting IgA-mediated signals in neutrophils, monocytes, and macrophages to stimulate phagocytosis, production of free radicals, and secretion of cytokines. Neutrophils have the highest expression of FcαRI out of all cell types. Following FcαRI activation, neutrophils and endothelial cells interact to promote migration of neutrophils toward tumor cells. Upon arriving at the tumor site, they release IL-1β and tumor necrosis factor-α (TNF-α) to achieve anti-tumor effects. Co-culturing of tumor cells, neutrophils, and IgA result in significant changes in the cell morphology of tumor cells. This is associated with high LC3-II expression in autophagosomes, but cell apoptosis remains constant. These phenomena suggest that autophagy is the main mechanism by which activated neutrophils combat against tumor cells [46].

### 3.6. Natural Killer Cells (NK Cells)

NK cells are involved in clearing virus infection and tumor cells. NK cell exerts its anti-tumor effects by direct killing of tumor cells, induction of cell apoptosis, secretion of IFN-γ, and inhibition of tumor metabolism. Recent studies have demonstrated that NK cells can induce autophagy in tumor cells, which may facilitate their survival [47].

## 4. Tumor Autophagy and Immunity

Existing studies have found that autophagy is active in various tumors, and can provide tumor-specific antigens for immune responses. T cells are arguably the most important cells in adaptive immunity, as they are required for almost all adaptive immune responses. T cells must be activated by interacting with a professional APC presenting an antigen which their T cell receptor recognizes before they can divide and perform their function. T cells themselves, however, can only function when activated to become effector cells. In addition, it provides the ATPs required for anti-tumor T-cells to activate antigen presenting cells (APCs). Aggregation of T-cells and APCs will induce the formation of inflammasomes, which promote IL-1β secretion and activation of CD8^+^ T-cells, eventually leading to cell-mediated anti-tumor immune responses [48,49,50]. For example, under oxidative stress, damage-associated molecular patterns (DAMPs) produced by tumor cells are phagocytosed by autophagosomes in tumor cells, which can be processed into cross-antigens [51,52]. Therefore, the autophagosomes in tumor cells can be used as carriers for tumor-specific cross antigens. In addition, puromycin-sensitive aminopeptidase (PSA) from tumor cells can be processed through self-autophagy to generate immunogenicity; recognition of processed PSA can result in activation of CD8^+^ T-cells and other anti-tumor immune responses [53] (Figure 1a).

Several lines of evidence highlight that autophagy modulates both the activity of immune effectors and the response of tumor cells to these effectors. In the following section, we will summarize the effect of autophagy activation how leads to the emergence of resistant tumor cells able to outmaneuver an effective immune response and escape from immune cell killing [54,55].

### 4.1. Autophagy and Tumor Escape

When conditional knockout of the *RB1CC1* gene was performed in a polyomavirus middle T-antigen (*PyMT*) oncogene driven mouse mammary tumor model, it was observed that inhibition of autophagy not only affects energy metabolism and cell proliferation, but also increases the anti-tumor immune surveillance capacity of the host, thereby preventing tumor growth [56]. Knockout of the *RB1CC1* gene elicited a drastic increase in tumor-infiltrating CD8^+^ T-cells in mice. When antibodies were used to deplete CD8^+^ T-cells in this model, tumor formation was accelerated [57]. This study showed that autophagy plays an important role in disrupting anti-tumor immunity. Other studies also showed that hypoxia-induced autophagy can resist the killing effects of T-cells in lung cancer [58,59]. Based on previous studies, it is evident that autophagy can disrupt tumor immune surveillance or anti-tumor immunity, and further promote continued tumor growth. Moreover, when tumor cells undergo autophagy, they can regulate tumor-specific immune responses by releasing immune regulatory factors. The recombinant anti-tumor, which is composed of epidermal growth factor (EGF) and the catalytic domain of the diphtheria toxin, was found to induce autophagy in malignant glioma cells to generate anti-tumor effects. At the same time, dying malignant glioma cells can release the immune regulatory factor HMGB1 to activate dendritic cells, thereby activating specific anti-tumor immune responses [60] (Figure 1c).

### 4.2. Autophagy and Tumor Immune Response

Immune surveillance in organisms functions to distinguish and remove mutated cells in order to prevent the development of tumors [49]. Mutated tumor cells will present specific antigens, which are recognized by the immune surveillance system in the human body; responses are then generated to clear the tumor cells [61]. The immune surveillance system mainly exerts its effects through immunity, which involves cells such as NK cells, natural killer T (NKT) cells, and helper T-cells [62]. However, despite this complex cross-check system, tumors can still develop within the body. Again, autophagy is a key player during this process. Cellular autophagy can promote the differentiation and maturation of immune cells, and acts to maintain internal homeostasis in immune cells. However, autophagy can be a deterrent for anti-tumor responses, and aid tumor cells in escaping from immune surveillance, resulting in continued growth and development of tumors [63]. Dying tumor cells can release ATP through exocytosis, which results in activation of immune cells. Drugs that inhibit autophagy inhibit ATP release, thereby hindering the development of immunogenicity. Similarly, deficiencies in autophagy-related genes such as *Atg5*, *Atg7*, *Atg10*, *Atg12*, *Beclin1*, lysosome-associated membrane protein 2 (*LAMP2*), and *VPS34* also results in down-regulation of immune responses [64,65]. Defects in autophagy lead to reductions in ATP release by tumor cells, and as a result, recruitment of monocytes, macrophages, dendritic cells may be insufficient to produce the required anti-tumor immune responses [32]. Animal experiments have shown that when autophagy is induced by chemotherapy, fluorescence intensity of ATP was greater in the control group as compared with that of *Atg5* liver–specific knockout mice [66] 18. These observations show that autophagy induced by chemotherapy do not mediate ATP release and recruitment of cytotoxic T-cells and dendritic cells to stimulate anti-tumor responses [46,67]. However, at the same time, immune-mediated cytotoxic effects may be inhibited by autophagy.

Solid tumor growth determinates the formation of a hypoxic microenvironment in the inner tumoral region due to inadequate oxygen diffusion. Hypoxia is a common characteristic of solid tumors. Autophagy can be activated in response to hypoxia as a survival mechanism in conditions of nutrient deprivation [68]. For example, hypoxia-induced autophagy in lung cancer cells can inhibit T-cell mediated cytotoxic effects [61,69]. On the other hand, inhibition of autophagy due to knockdown of *Atg* and *Beclin-1* genes can reverse T-cell mediated cytotoxic effects. Hypoxia-induced autophagy can affect tumor cell killing by NK-cells through selective degradation of synaptic connexin 43. In addition, inhibition of the PI3K signaling pathway increases sensitivity of tumor cells to NK cells. Inhibition of the autophagy-related protein Atg7 can increase the anti-tumor effects of CD8^+^ T-cells, preventing the formation of tumors in the gastrointestinal tract [70]. Overall, these results show that although autophagy can promote anti-tumor immune responses, it can also inhibit immune cell activities to facilitate evasion of tumor cells from the immune system.

## 5. Targeting Autophagy in Cancer Therapy

The relationship between autophagy and tumors is bidirectional; autophagy has both inhibitory and stimulating effects on tumors. Under normal physiological conditions, ROS produced from damaged organelles can induce DNA damage. This chromosomal instability may eventually lead to tumor development [7]. On the other hand, autophagy also removes damaged organelles to maintain normal cellular structure and chromosomal stability [71]. Therefore, some researchers have proposed that autophagy can inhibit the development of tumors. However, some studies have demonstrated that when the autophagy-related gene was knocked out in mice, the prevalence for tumors following the addition of carcinogens was higher in autophagy-deficient mice as compared with that in wild type mice. This was also associated with reduced time to tumor development [72]. In tumor cells, cellular autophagy is inhibited by multiple factors. This may accelerate DNA damage, leading to disruptions in gene integrity and accumulation of deleterious mutations, which eventually leads to tumor formation, and in serious cases, development of malignancies [66]. On the other hand, autophagy can degrade misfolded proteins and damaged organelles in tumor cells, and provide nutrients and energy to tumor cells, thereby preventing apoptosis [73]. In addition, autophagy can induce resistance against chemotherapeutic agents, resulting in formation of chemo-resistant tumor cells [74]. Studies have shown that following treatment of antineoplastic agents and X-rays in tumor cells, the number of autophagosomes in the cell was increased, which was correlated with increased ability of tumor cells to survive [75,76].

A dominant feature of tumor cells is their ability to effectively evade apoptosis; this is one of the most difficult problems in cancer therapy. Recent studies have shown that autophagy plays an important role during chemotherapy. Before tumor cells can develop a rich vascular network, nutrients produced via autophagy can be used to supply energy. In recent years, discovery of anti-tumor targets has allowed cancer therapies to progress from traditional cytotoxic drugs to new and specific anti-tumor drugs [77]. In addition, as shown in human lymphoma cells, autophagy inhibitors can increase then sensitivity of tumor cells to apoptosis-inducing agents. Due to the dual relationships between tumors and autophagy, it is evident that simple inhibition or induction of autophagy will not be effective in cancer therapies [78,79]. Instead, targeted anti-tumor drugs may be required for effective tumor therapy. How do we develop anti-cancer drugs to induce autophagy and kill tumor cells, and how do we inhibit the tumor-promoting role of autophagy while enhancing its tumor suppression effects? All these questions need to be answered.

As autophagy has a demonstrated role in promoting tumor initiation, growth, survival, maintenance, malignancy, and metastasis in varying settings, therapeutic targeting of autophagy for cancer therapy may have value. Efforts in the pharmaceutical academia have focused on the development of small molecule targeting the components of the autophagy pathway (Table 2).

## 6. Conclusions

Surgery, radiotherapy, and chemotherapy are the three major traditional methods for cancer treatments. However, these methods cause significant adverse effects in patients and are not effective against all tumors. The rapid development and overlap between immunology, molecular biology, and other related disciplines is expected to improve tumor immunotherapy, where treatment results in minimal damage to host cells but can effectively control tumor growth and metastasis. In the tumor microenvironment, the interactions between tumor cells and immunity have major effects on tumor cell growth. On one hand, cytokines, immunoglobulins, and immune-related cells in the tumor microenvironment regulate autophagy in tumor cells. On the other hand, autophagy in tumor cells can affect immune responses inside the tumors. In addition, autophagy in tumor cells play dual roles in tumor development. However, the specific molecular mechanisms involved in these processes still require further investigation. Tumor immunotherapy mainly includes active, passive, and adoptive immunotherapies. The key is to use a combination therapy approach to increase the immune responses in biological systems to effectively treat tumors. Basic and clinical research in tumor immunotherapy is currently a much-studied area of research. In order to further our understanding of the tumor microenvironment and generate integrated cancer therapies, more research needs to be performed in this rapidly developing research fields.

## Figures and Tables

**Table 1:** Autophagy-related genes.

Yeast	Mammals	Gene Functions
Atg1	ULK1,2	Protein kinase: Atg1–Atg13–Atg17–Atg29 complex
Atg2	Atg2	Atg2–Atg18 complex
Atg3	Atg3	E2-like enzyme
Atg4	Atg4	Hydrolases: Atg8 activation
Atg5	Atg5	E3-like enzyme for Atg5–Atg12 conjugation
Atg6	Beclin-1	Subunit of Vps34/PI3K complex
Atg7	Atg7	E1-like enzyme for LC3-conjugation
Atg8	LC3	Ubiquitin-like modifiers: Conjugates to PE to localize to autophagosome
Atg9	Atg9	Atg9 interacts Atg2–Atg18 complex: membrane bound
Atg10	Atg10	E2-like enzyme for Atg12-conjugation
Atg12	Atg12	Modifier: Conjugates to Atg5
Atg13	Atg13	mTOR signaling: Atg1–Atg13–Atg17–Atg29 complex
Atg14	Atg14	Subunit of Vps34 PI3K complex
Atg16	Atg16	E3-like activity
Atg17	RB1CC1	Regulator: Atg1–Atg13–Atg17–Atg29 complex complex
Atg18	WIPI-1	Atg2–Atg18 complex

**Table 2:** Therapeutic compounds and targets that modulate autophagy-dependent immune responses.

Drugs	Cancer Types	Autophagy-Modulating Mechanism	Reference
Honokiol	Prostate cancer	Induce ROS-dependent autophagy cytoprotectively	[80]
Tamoxifen	Breast cancer	Down-regulate activity on anti-oxidative enzyme	[81]
2-Methoxyestradiol	Osteosarcoma	Induce RNA-dependent protein kinase (PKR)-dependent autophagy	[82]
Temozolomide	Glioma	Down-regulate expression on activating transcription factor 4 (ATF4)	[83]
Oridonin	Esophageal cancer	Targeting epidermal growth factor (EGF) interactions in ROS dependent mechanism	[84]
Cucurbitacin	Lung cancer	Induced protective autophagy mediated by ROS	[85]
Chloroquine	Bladder cancer	Targeting lysosomal functions and block autophagy	[86]
Quercetin	Cervical cancer	Down-regulate activity on LC-3 and beclin-1	[87]
Eriocalyxin B	Breast cancer	Suppression of Akt/mTOR/p70S6K signaling	[88]
Shikonin	Liver cancer	Targeting extracellular signal–regulated kinases (ERK) interactions in ROS dependent mechanism	[89]

## Abbreviations

4EBP	eIF4E binding protein
Akt	Protein kinase B
AMPK	AMP-activated protein kinase
APCs	Antigen presenting cells
ATF4	Activating transcription factor 4
ATP	Adenosine triphosphate
Atg	Autophagy-related genes
Bcl-2	B-Cell lymphoma 2
BH3	Bcl-2 homology 3
BRCA1	Breast cancer 1
CD89	IgA Fc receptor FcαRI
CMA	Chaperone-mediated autophagy
COX-1	Cyclooxygenase
DAMPs	Damage-associated molecular patterns
DT-EGF	Diphtheria toxin-epidermal growth factor
EGF	Epidermal growth factor
ER	Endoplasmic reticulum
ERK	Extracellular signal–regulated kinases
GMCSF	Granulocyte-macrophage colony-stimulating factor
HMGB1	High mobility group box 1
IFN-γ	Interferon-γ
IL-1R	IL-1 receptors
IL-1β	Interleukin 1 β
IκB	Inhibitor of κ B
LAMP2	Lysosome-associated membrane protein 2
LC3	Light chain 3
MIF	Macrophage migration inhibitory factor
MIp1α	Macrophage inflammatory protein 1 α
rMIF	Recombinant MIF
NF-κB	Nuclear factor κ-light-chain-enhancer of activated B cells
NK	Cells natural killer cells
PE	Phosphatidylethanolamine
PKR	RNA-dependent protein kinase
PI3K	Phosphatidylinositol-4,5-bisphosphate 3-kinase
PIKK	Phosphatidylinositol 3 kinase-related kinase
PSA	Puromycin-sensitive aminopeptidase
RAGE	Receptor for advanced glycation end products
RANTES	Regulated on activation, normal T cell expressed and secreted
RB1CC1	Inducible coiled-coil 1
ROS	Reactive oxygen species
SAPK/JNK	Stress-activated protein kinase /c-Jun N-terminal kinases
TGF-β	Transforming growth factor β
TNF-α	Tumor necrosis factor-α
mTOR	Mammalian target-of-rapamycin
mTORC1	Mammalian target-of-rapamycin complex 1
ULK1	Unc-51 like autophagy activating kinase 1
VPS34	Vacuolar protein sorting 34
